# 

**Published:** 1906-10

**Authors:** 


### THE AMERICAN
### Journal of Dental Science

The Official Organ of the Florida, Georgia and South Carolina State Dental Societies.

**THIRD SERIES.**

**VOLUME XXXVII. OCTOBER, 1906. NO. 10.**

EDITORS:

WM. GIRD BEECROFT, D. D. S., Madison, Wis. B. H. TEAGUE, D. D. S., Aiken, S. C.

Published on the 10th day of each month

Subscription Price, $1 00 per year. Foreign countries, $1.50 per year.

*Entered as Second-Class Matter, Madison, Wis.*

Address all communications, both business and editorial, to

The Dental Publishing Company, Madison, Wisconsin.

